# RETRACTED: Lee et al. Lemon Balm Extract ALS-L1023 Regulates Obesity and Improves Insulin Sensitivity via Activation of Hepatic PPARα in High-Fat Diet-Fed Obese C57BL/6J Mice. *Int. J. Mol. Sci.* 2020, *21*, 4256

**DOI:** 10.3390/ijms26041696

**Published:** 2025-02-17

**Authors:** Dongju Lee, Yujin Shin, Jong Seong Roh, Jiwon Ahn, Sunhyo Jeoong, Soon Shik Shin, Michung Yoon

**Affiliations:** 1 Department of Biomedical Engineering, Mokwon University, Daejeon 35349, Republic of Korea; dlehdwn100@naver.com (D.L.); ujin2821@naver.com (Y.S.); jsh0227@mokwon.ac.kr (S.J.); 2 Department of Microbiology and Immunology, Pusan National University School of Medicine, Busan 50612, Republic of Korea; jsjs101@naver.com; 3 Genome Research Center, Korea Research Institute of Bioscience and Biotechnology, Daejeon 34141, Republic of Korea; jiwon@kribb.re.kr; 4 Department of Formula Sciences, College of Oriental Medicine, Dongeui University, Busan 47340, Republic of Korea

The journal retracts the article titled “Lemon Balm Extract ALS-L1023 Regulates Obesity and Improves Insulin Sensitivity via Activation of Hepatic PPARα in High-Fat Diet-Fed Obese C57BL/6J Mice” [1], cited above.

Following the publication, concerns were brought to the attention of the Editorial Office regarding the duplication of two figures found in this [1] and two other articles [2,3].

Adhering to our complaints procedure, the Editorial Office and Editorial Board conducted an investigation that confirmed that Figures 1d and 5a from this publication [1] overlap with Figure 7C in publication [2] and Figure 8d in the other article [3], respectively. While the authors cooperated during the investigation, they were unable to provide raw material for Editorial Board evaluation. Consequently, the Editorial Board lost confidence in the reliability of the findings and decided to retract this publication [1], as per MDPI’s retraction policy (https://www.mdpi.com/ethics#_bookmark30).

This retraction was approved by the Editor-in-Chief of the IJMS journal.

Michung Yoon agreed to this retraction. The remaining authors did not provide a comment on this decision.
